# Symptom Distribution Regularity of Insomnia: Network and Spectral Clustering Analysis

**DOI:** 10.2196/16749

**Published:** 2020-04-16

**Authors:** Fang Hu, Liuhuan Li, Xiaoyu Huang, Xingyu Yan, Panpan Huang

**Affiliations:** 1 College of Information Engineering Hubei University of Chinese Medicine Wuhan China; 2 College of Basic Medicine Hubei University of Chinese Medicine Wuhan China

**Keywords:** insomnia, core symptom, symptom community, symptom embedding representation, spectral clustering algorithm

## Abstract

**Background:**

Recent research in machine-learning techniques has led to signiﬁcant progress in various research ﬁelds. In particular, knowledge discovery using this method has become a hot topic in traditional Chinese medicine. As the key clinical manifestations of patients, symptoms play a signiﬁcant role in clinical diagnosis and treatment, which evidently have their underlying traditional Chinese medicine mechanisms.

**Objective:**

We aimed to explore the core symptoms and potential regularity of symptoms for diagnosing insomnia to reveal the key symptoms, hidden relationships underlying the symptoms, and their corresponding syndromes.

**Methods:**

An insomnia dataset with 807 samples was extracted from real-world electronic medical records. After cleaning and selecting the theme data referring to the syndromes and symptoms, the symptom network analysis model was constructed using complex network theory. We used four evaluation metrics of node centrality to discover the core symptom nodes from multiple aspects. To explore the hidden relationships among symptoms, we trained each symptom node in the network to obtain the symptom embedding representation using the Skip-Gram model and node embedding theory. After acquiring the symptom vocabulary in a digital vector format, we calculated the similarities between any two symptom embeddings, and clustered these symptom embeddings into five communities using the spectral clustering algorithm.

**Results:**

The top five core symptoms of insomnia diagnosis, including difficulty falling asleep, easy to wake up at night, dysphoria and irascibility, forgetful, and spiritlessness and weakness, were identified using evaluation metrics of node centrality. The symptom embeddings with hidden relationships were constructed, which can be considered as the basic dataset for future insomnia research. The symptom network was divided into five communities, and these symptoms were accurately categorized into their corresponding syndromes.

**Conclusions:**

These results highlight that network and clustering analyses can objectively and effectively find the key symptoms and relationships among symptoms. Identification of the symptom distribution and symptom clusters of insomnia further provide valuable guidance for clinical diagnosis and treatment.

## Introduction

### Background

Insomnia is a subjective complaint of a sleep disorder in which the patient has difficulty falling asleep or remaining asleep as long as desired. Insomniacs usually have low energy, less concentrating power, reduced appetite, and mood swings, leading to low performance throughout the day at work [1]. Approximately 16% of the population is reported to suffer from insomnia [2]. Clinical research has shown that traditional Chinese medicine (TCM) can be successfully applied in the treatment of insomnia [3,4]. However, the evaluation criteria of TCM diagnosis and treatment of insomnia remain unexplored. The most fundamental reason for this lack is that the clinical manifestations of insomnia are complicated and diverse; therefore, TCM physicians have difficulties in accurately extracting the core symptoms to carry out effective treatment according to clinical characteristic categories.

Machine learning, a subset of artiﬁcial intelligence and a data-oriented approach, has attracted substantial attention from various domains [5,6]. Researchers have already proposed a huge number of algorithms and models referring to machine learning to discover the hidden relationships between entities from different research ﬁelds [7,8]. TCM datasets have characteristics of “big data,” particularly with respect to the complex relationships among diseases, syndromes, symptoms, prescriptions, herbs, diagnosis, and treatment [9]. As the key clinical manifestations of patients, symptoms play a significant role in clinical diagnosis and treatment, which evidently have their underlying TCM mechanisms. There are frequently multiple interrelated symptoms under the same subgroup. A symptom network reflects the macroscopic law of the dynamic process of complex symptoms under the influence of certain driving forces. In recent decades, several researchers have applied various machine-learning approaches to discover the potential regulations for treating insomnia. Ahuja et al [10] applied 15 machine-learning algorithms and took 14 leading factors into consideration for predicting insomnia. The results of this analysis showed that insomnia primarily depends on vision problems, mobility problems, and sleep disorder. Park et al [11] developed 3 prediction models for sleep quality using machine-learning techniques to uncover the relationships between sleep quality and sleep-related factors. The results suggested that morning activity, and exposure to total and outside light during daytime are important contributors to sleep quality. Based on the Bayesian belief network model, Seixas et al [12] assessed the sleep duration and physical activity proﬁles that provided the lowest diabetes prevalence among black and white subjects. Hu et al [13,14] discovered the core symptoms and symptom distribution rule of insomnia using a network analysis method. Li et al [15] explored suitable preprocessing methods for analysis of TCM clinical data based on a prospective study on patients with insomnia treated according to syndrome differentiation. Weng et al [16] determined the frequency of each herb and association rules among the herbs for insomnia using data mining methods.

With continuous development of artificial intelligence, heterogeneous information network [17] and graph embedding [18] can be conducted to construct a medical network and train the various medical node embeddings for in-depth analysis of TCM data, including analysis of the molecular mechanisms of symptoms [19], herb target prediction [20], and disease comorbidity patterns [21]. Yang et al [22] proposed a heterogeneous network embedding representation algorithm to construct a heterogeneous symptom-related network, which was applied to obtain the low-dimensional vector representation of symptom nodes. This model was used to predict disease genes with high performance and obtained better results than other well-known disease gene prediction algorithms. Wang et al [20] presented an herb target interaction network approach for novel herb target prediction mainly relying on symptom-related associations. The above studies helped to effectively discover the relationships among disease mechanisms, symptoms, herbs, targets, ingredients, genes, and related factors; however, the critical factors of syndrome differentiation and treatment, and their corresponding relationships require further study. In particular, the most effective methods for exploring the key factors and relationships in TCM data, and to support the clinical diagnosis and treatment remain unclear.

### Objectives

In this study, we explored the potential regularity of symptoms for diagnosing insomnia using complex network and machine-learning approaches. After constructing the symptom network with speciﬁc criteria, we identiﬁed the most important symptom nodes using four node importance evaluation metrics. Using the node-embedding technique [23,24], we acquired each symptom node embedded in the symptom network, and constructed the speciﬁc symptom vocabulary with the digital formation of vectors. Further, we divided the symptoms into several communities through similarity calculations between any two symptom embeddings using the spectral clustering algorithm. Finally, we obtained the core symptoms and symptom clusters, and then summarized the symptom distribution rule of insomnia. Compared to previous studies, we combined the complex network with a machine-learning approach to find the key symptoms and their corresponding symptom distribution rule. This study will provide a novel exploratory analysis method to discover clinically relevant information from TCM data.

## Methods

### Data Extraction

The analysis dataset of insomnia was extracted from the hospital information system at Guo Yi Tang Affiliated Hospital of Hubei University of Chinese Medicine (Wuhan, Hubei, China). The inclusion criteria for record selection were patients diagnosed with typical symptoms of insomnia (sleep disorder is the main symptom and the other symptoms are secondary to insomnia), aged 14-70 years, and insomnia occurring between 1 month and 30 years. The exclusion criteria were noncollaborators, including those unable to adhere to treatment or any noncompliance that would affect data collection and efficacy evaluation, and pregnant women or terminally ill patients.

Based on these criteria, we extracted 807 effective outpatient electronic medical records (EMRs) as the research data. Through analyzing the theme data, we cleaned the raw data and selected some signiﬁcant features, including syndromes and their corresponding symptoms, and then formed the analysis dataset of insomnia.

### Steps of Data Processing

**Figure 1:**
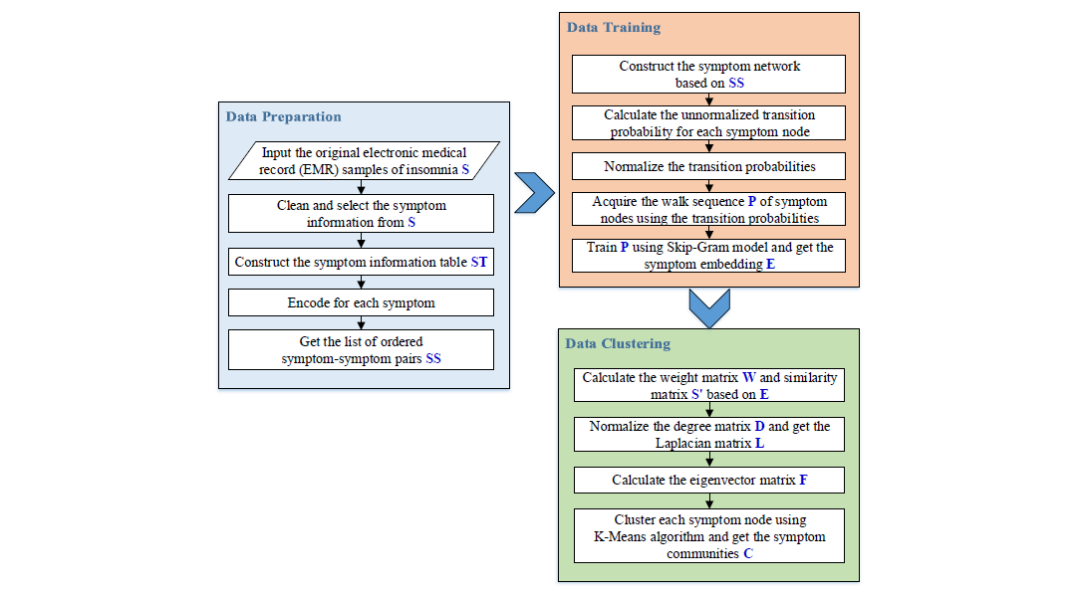
Flowchart of data processing.

A summary of the data processing for insomnia is outlined in Figure 1. We divided the data processing into three steps: data preparation, data training, and data clustering.

In the ﬁrst step, we obtained the original EMRs dataset *S* from the hospital information system, cleaned and selected the symptom information from *S*, and then constructed the symptom information table *ST.* After encoding each symptom, the list of ordered symptom-symptom pairs *SS* was acquired.

In the second step, we constructed the symptom network based on *SS,* calculated the transition probability for each symptom node, and normalized the probabilities to acquire the walk sequence *P* of symptom nodes. After training *P* based on the Skip-Gram model [25], we obtained the symptom embeddings *E.*

In the third step, we calculated the weight matrix *W* and similarity matrix *S′* based on the symptom embeddings *E.* From the degree matrix *D* and the Laplacian matrix *L*, we obtained the eigenvector matrix *F.* After clustering *F* using the K-means algorithm, the symptom communities *C* were acquired.

### Construction of the Symptom Network Model

Based on complex network theory [26,27], we constructed the insomnia symptom network *G(V,E)*, where *V* is the node set of symptoms and *E* denotes the edge set between any two symptoms. The rules of symptom network construction were as follows: each symptom in the records was considered a node in the network, the connection between any two symptoms co-occurring in the same diagnosis was considered an edge, and the weight of an edge was considered as the co-occurrence frequency of any two symptoms.

**Figure 2:**
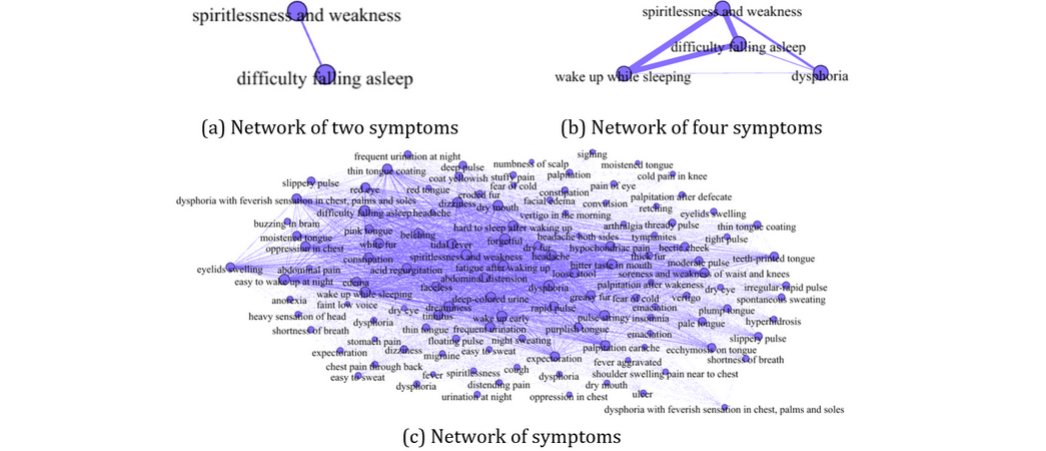
Construction process of the symptom network.

The construction process of the insomnia symptom network based on these rules is schematically outlined in Figure 2. As shown in Figure 2a, we constructed a network with two symptom nodes, *spiritlessness and weakness* and *difficulty falling asleep*, and denoted an edge representing these two symptoms co-occurring in the same diagnosis. During development, two other symptom nodes, *wake up while sleeping* and *dysphoria*, and their corresponding weighted edges were added to the network, as shown in Figure 2b. Finally, we acquired an undirected and weighted symptom network of insomnia including 164 nodes and 10,244 edges, as shown in Figure 2c.

### Evaluation Metrics of Node Centrality

For complex networks, several evaluation metrics of node centrality are typically used to identify the core nodes [28]. The representative metrics include degree centrality, closeness centrality, betweenness centrality, and eigenvector centrality, which can reﬂect the node centrality (also called node importance) from different aspects. Degree centrality reﬂects the direct inﬂuence and the acquiring information ability of one node [29], closeness centrality reflects the distance properties between one node and other nodes [29], betweenness centrality measures the proportion of the shortest paths through one node [29], and eigenvector centrality represents the importance of one node comprehensively considering the importance of its neighbor nodes [30]. The equations of these four evaluation indices are as follows:

Degree centrality: 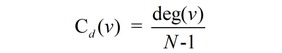


Betweenness centrality: 
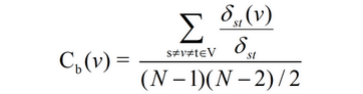


Closeness centrality: 
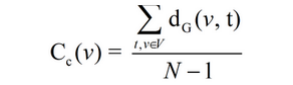


Eigenvector centrality: 
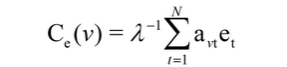


The complex network is denoted as *G(V,E),* where *V* is the set of nodes and *E* is the set of edges. In the equation of degree centrality, *deg* (*v*) is the degree of node *v* and *N* is the number of nodes. In the betweenness centrality, *δ_st_* is the number of the shortest paths from node *s* to node *t*, and *δ_st_ (v)* is the number of shortest paths through node *v*. In the closeness centrality equation, *d_G_(v,t)* is the shortest path from node *v* to node *t.* In the eigenvector centrality, *A* represents the adjacent matrix of a network; if there is an edge between node *v* and node *t*, *a_vt_*=1, otherwise *a_vt_*=0. *λ_1_*, *λ_2_*,…, *λ_N_* are the eigenvalues of *A*, and *e_t_* is the eigenvector of *λ_t_*.

### Pearson Correlation Coefficients of Symptoms

The Pearson correlation coefficient, sometimes called the Pearson product-moment correlation coefficient, is a measure of the linear correlation between two variables [31,32]. It has a value between –1 and +1, where +1 indicates a complete positive linear correlation, 0 is no linear correlation, and –1 is a complete negative linear correlation. The deﬁnition of Pearson correlation coefficient *r* is as follows:


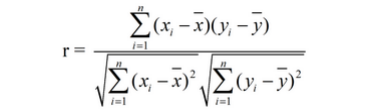


where *n* is the sample size; *x_i_* and *y_i_* are the individual sample points indexed with *i*; 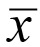 is the sample mean represented as:

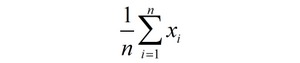



and analogously for 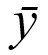.

**Figure 3:**
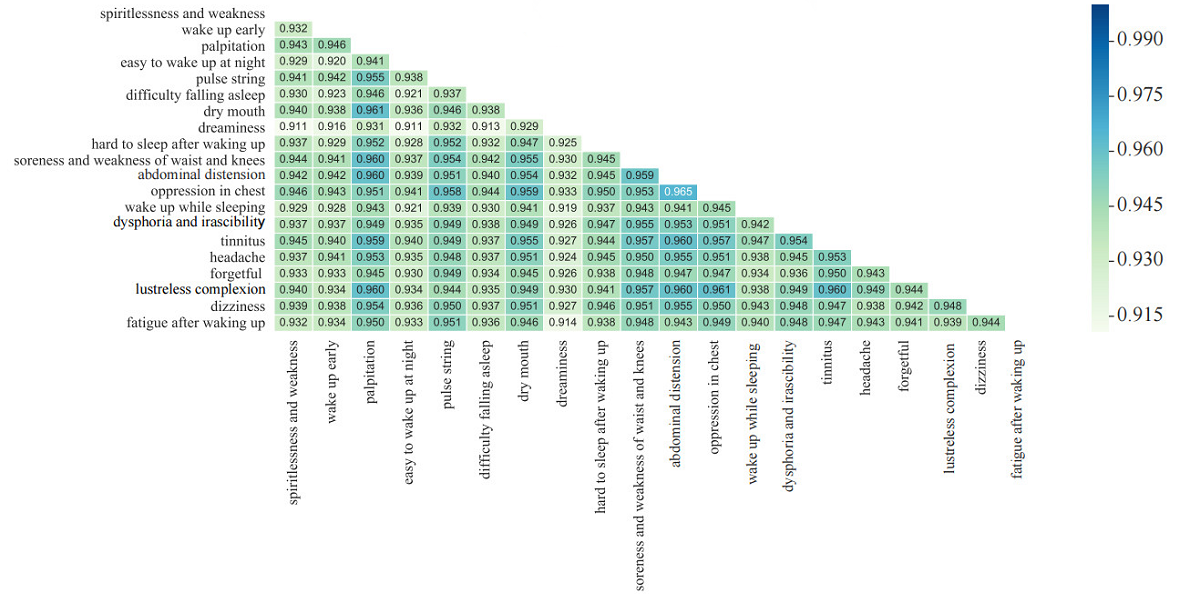
Pearson correlation coefficients between any two symptoms.

We calculated the Pearson correlation coefficients between any 2 of the top 20 core symptom nodes from the symptom network. The relative heatmap is provided in Figure 3, in which the strengths of correlation values are represented using different color shading.

### Training the Symptom Embeddings

**Figure 4:**
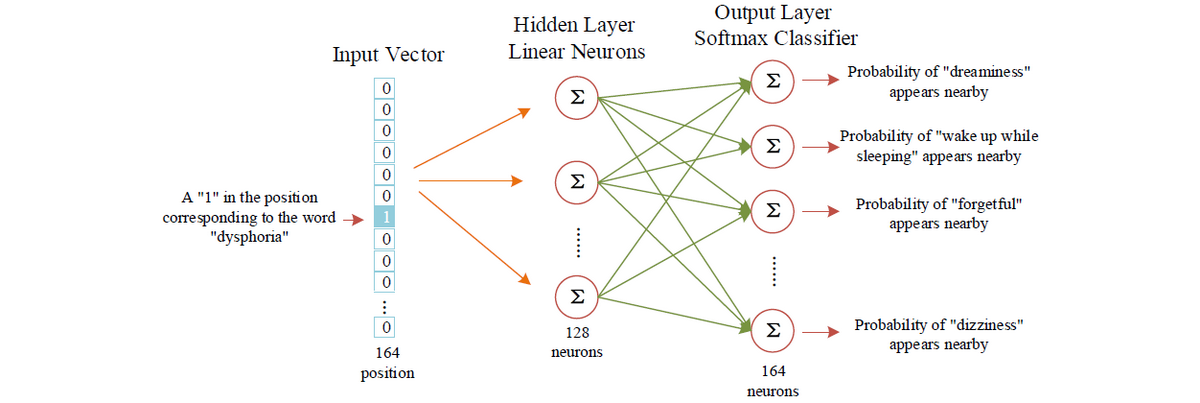
Skip-Gram model of symptoms.

Based on the matrix of the symptom network, we use the Skip-Gram model [25] to train the insomnia symptom embeddings (also called symptom vectors). We ﬁrst built a vocabulary of 164 insomnia symptom terms. We represent an input symptom term such as *dysphoria* as a one-hot vector. This vector will have 164 components (one for every symptom in our vocabulary), and we placed “1” in the position corresponding to the symptom *dysphoria* and “0*”* in all other positions. The output of the network is a single vector containing 128 components. For each symptom in our vocabulary, the probability of randomly selecting a nearby symptom was calculated. The neural network model for training the symptom embeddings is outlined in Figure 4. In this model, we set the input layers as the 164 one-hot symptom vectors, the number of neurons in the hidden layer as 128, and the activation function in the output layer as the softmax function. Therefore, when evaluating the trained network on an input symptom one-hot vector, the output vector will be a probability distribution (ie, a series of ﬂoating point values rather than a one-hot vector). Consequently, we can obtain the probabilities of the symptoms such as *dreaminess, wake up while sleeping, forgetful,* and *dizziness* appearing close to the symptom *dysphoria* in the network*.*

**Figure 5:**
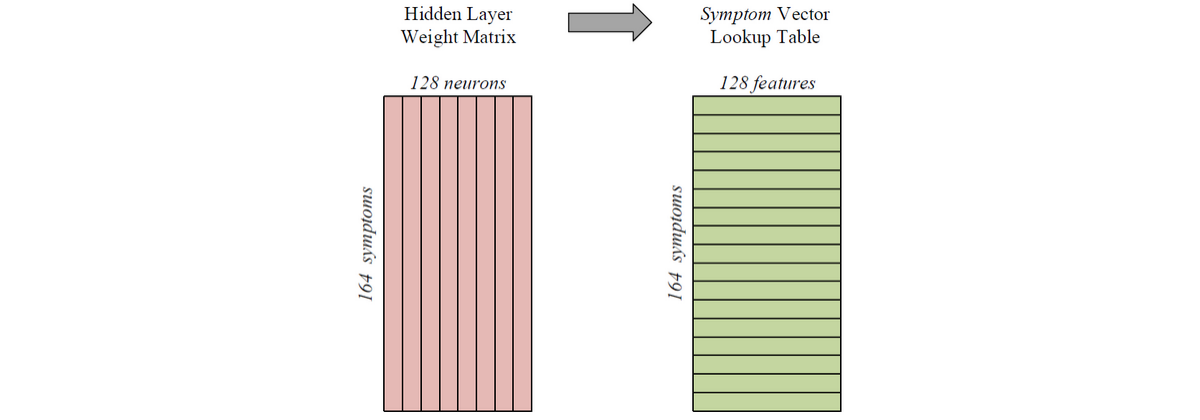
Representation of symptom embeddings.

After training the model as shown in Figure 4, we acquired the weight matrix (ie, the symptom embeddings with 128 features) in the hidden layer. This weight matrix has 164 rows (one for each symptom in our vocabulary) and 128 columns (one for every hidden neuron). The symptom embedding lookup table is obtained from the weight matrix in the hidden layer as shown in Figure 5.

### Clustering the Symptom Embeddings

To ﬁnd the rule of symptom distribution and the symptom clusters of insomnia, we used the spectral clustering algorithm [33,34]—as a representative community detection algorithm used in complex networks—to divide the symptom network with 164 nodes and 10,244 edges into real communities. A community comprises one group or cluster of nodes in which the links between nodes are densely connected to each other but are sparsely connected with other communities [35].

We calculated the similarity values between any two symptom embeddings and divided the symptoms with high similarity values into the same community. The clustering process is as follows: we constructed the weight matrix *W* (ie, similarity matrix) through calculating the specific distance between two arbitrary symptom nodes *v_i_* and *v_j_*, obtained the degree matrix *D,* calculated the Laplacian matrix (*L*=*D*–*W*), and obtained the normalized Laplacian matrix *L′.* We then found the ﬁrst *k* minimum eigenvalues and their corresponding eigenvectors of *L′*, and constructed the eigenmatrix *F* using these eigenvectors. *F* was clustered using the K-means algorithm to finally acquire the symptom clusters of insomnia.

## Results

### Core Symptom Analysis

**Figure 6:**
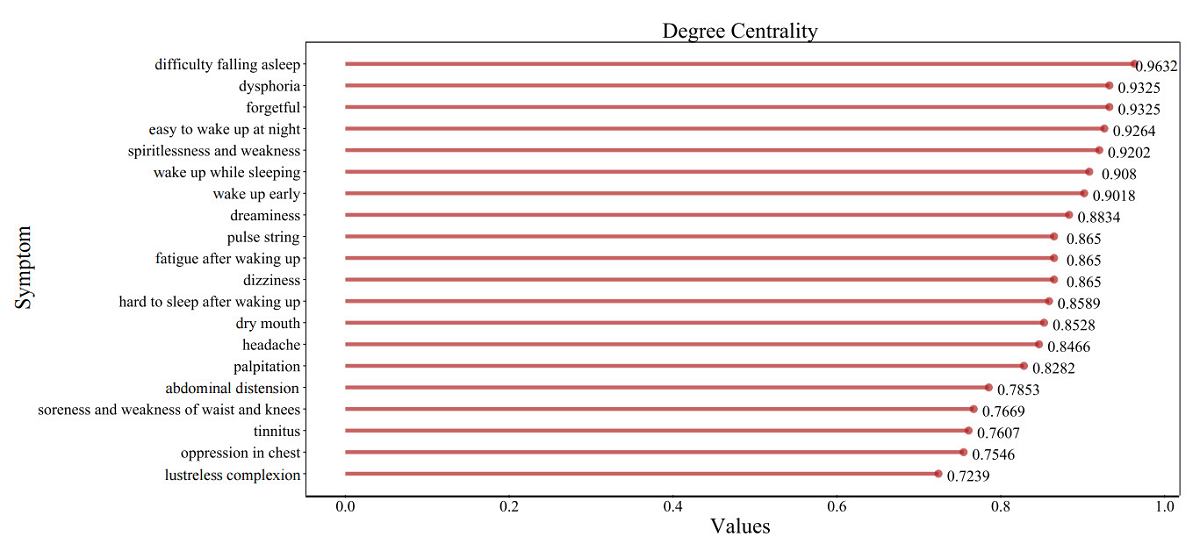
Degree centrality of symptoms.

**Figure 7:**
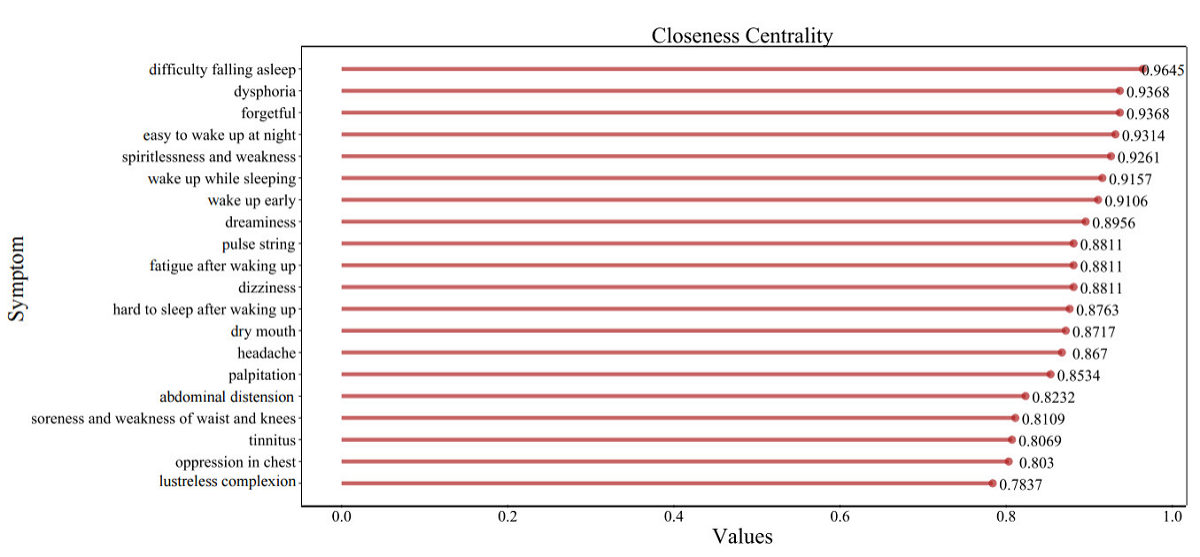
Closeness centrality of symptoms.

**Figure 8:**
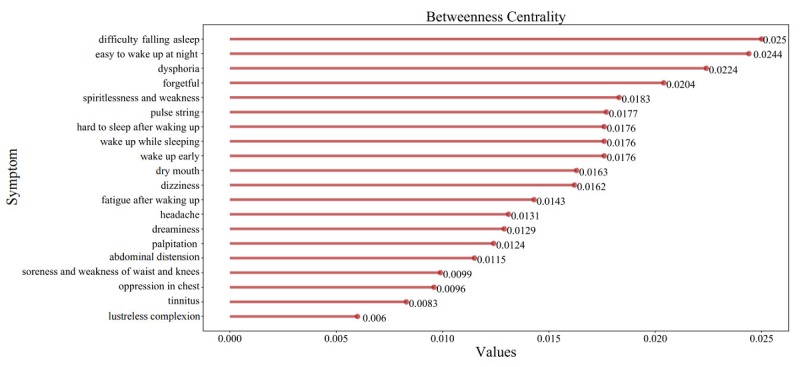
Betweenness centrality of symptoms.

**Figure 9:**
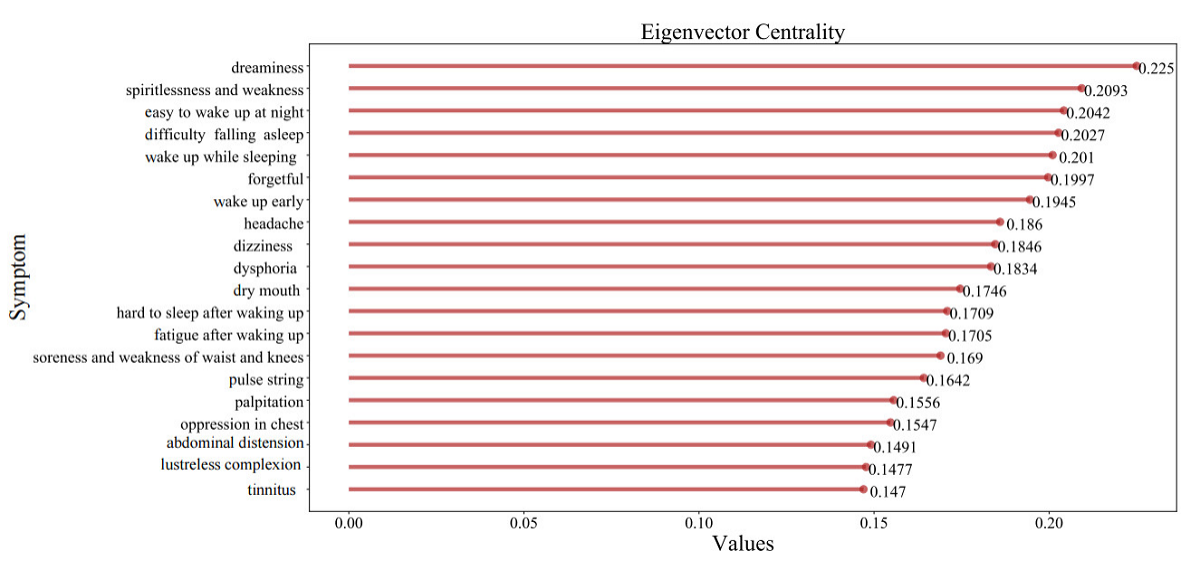
Eigenvector centrality of symptoms.

We used four evaluation metrics to calculate the different centrality values of each node in the symptom network, and display the top 20 signiﬁcant symptoms of 164 nodes in Table 1. The plots for degree centrality, closeness centrality, betweenness centrality, and eigenvector centrality are presented in Figure 6, 7, 8, and 9, respectively. The signiﬁcant symptoms calculated by these four approaches were nearly identical. In particular, the degree centrality, closeness centrality, and betweenness centrality identiﬁed the same top 5 core symptoms, including *difficulty falling asleep, easy to wake up at night, dysphoria and irascibility, forgetful,* and *spiritlessness and weakness.* The eigenvector centrality found the same 3 symptoms *difficulty falling asleep, easy to wake up at night,* and *spiritlessness and weakness*, and could also find two other symptoms *wake up while sleeping* and *dreaminess*. Therefore, based on the symptom network of insomnia, the core symptoms can be identiﬁed accurately using these evaluation metrics referring to multiple aspects.

**Table 1:** Node centrality analysis of the symptom network^a^.

No.	Symptoms	Degree	Closeness	Betweenness	Eigenvector
1	difficulty falling asleep	0.9632^b^	0.9645^b^	0.025^b^	0.2027^b^
2	forgetful	0.9325^b^	0.9368^b^	0.0204^b^	0.1997
3	dysphoria and irascibility	0.9325^b^	0.9368^b^	0.0224^b^	0.1834
4	easy to wake up at night	0.9264^b^	0.9314^b^	0.0244^b^	0.2042^b^
5	spiritlessness and weakness	0.9202^b^	0.9261^b^	0.0183^b^	0.2093^b^
6	wake up while sleeping	0.908	0.9157	0.0176	0.201^b^
7	wake up early	0.9018	0.9106	0.0176	0.1945
8	dreaminess	0.8834	0.8956	0.0129	0.225^b^
9	dizziness	0.865	0.8811	0.0162	0.1846
10	fatigue after waking up	0.865	0.8811	0.0143	0.1705
11	pulse string	0.865	0.8811	0.0177	0.1642
12	hard to sleep after waking up	0.8589	0.8763	0.0176	0.1709
13	dry mouth	0.8528	0.8717	0.0163	0.1746
14	headache	0.8466	0.867	0.0131	0.186
15	palpitation	0.8282	0.8534	0.0124	0.1556
16	abdominal distension	0.7853	0.8232	0.0115	0.1491
17	soreness and weakness of waist and knees	0.7669	0.8109	0.0099	0.169
18	tinnitus	0.7607	0.8069	0.0083	0.147
19	oppression in chest	0.7546	0.803	0.0096	0.1547
20	lusterless complexion	0.7239	0.7837	0.006	0.1477

^a^The top 20 symptoms are ranked in order of importance.

^b^The top 5 most important values in each column.

### Symptom Correlation Analysis

Based on Figure 3, strong correlations were identified between any two of the top 20 symptoms with a range of 0.91 to 0.97. The correlation coefficient between *oppression in chest* and *abdominal distension* was 0.97, denoting that these two symptoms have the strongest correlation. A correlation coefficient of 0.96 was obtained between pairs of the following symptoms: *palpitation* and *soreness and weakness of waist and knee*, *pulsing string, dry mouth, abdominal distension*, *tinnitus, lusterless complexion; pulsing string* and *oppression in chest*, *tinnitus, lusterless complexion, soreness and weakness of waist and knee, abdominal distension; abdominal distension* and *tinnitus, lusterless complexion, dizziness; oppression in chest* and *tinnitus, dry mouth, lusterless complexion;* and *tinnitus* and *lusterless complexion*. These results indicate that there are strong correlations between these symptoms for the clinical diagnosis of insomnia.

### Symptom Clustering Analysis

To obtain the best result of symptom distribution, we trained the symptom embeddings using the different embedding dimensions *d*=128 and *d=*164 in the node-embedding model and divided the symptom network into different communities by changing the cluster numbers (*c*=4 and *c*=5) in the spectral clustering algorithm.

**Figure 10:**
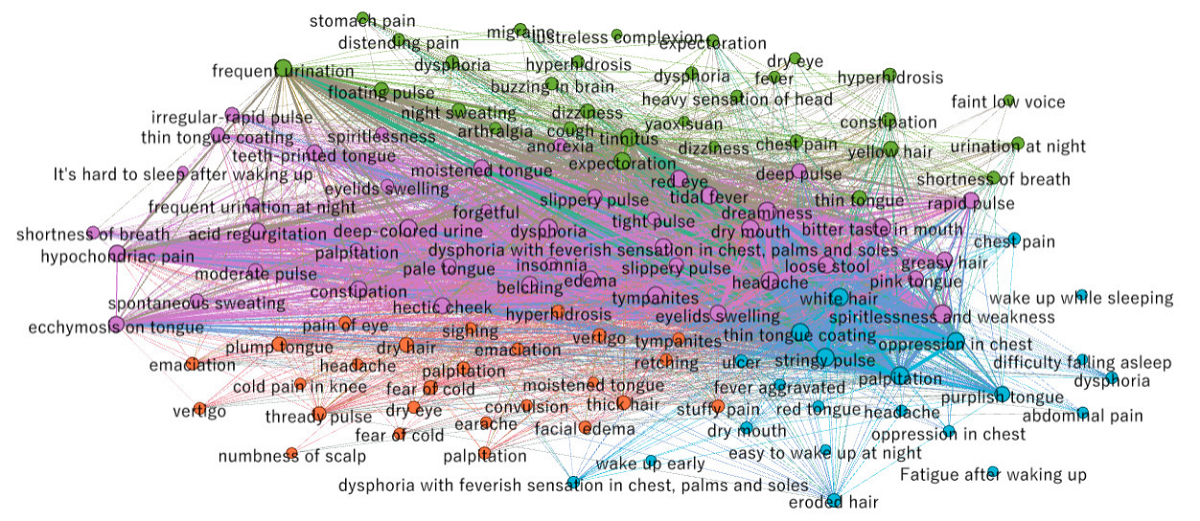
Symptom communities (d=128 and c=4).

**Figure 11:**
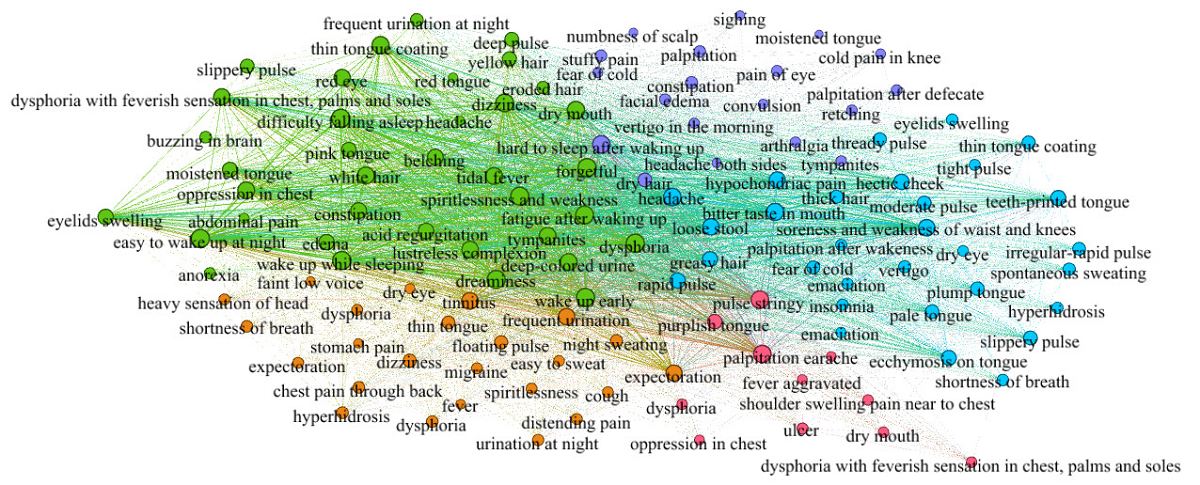
Symptom communities (d=128 and c=5).

**Figure 12:**
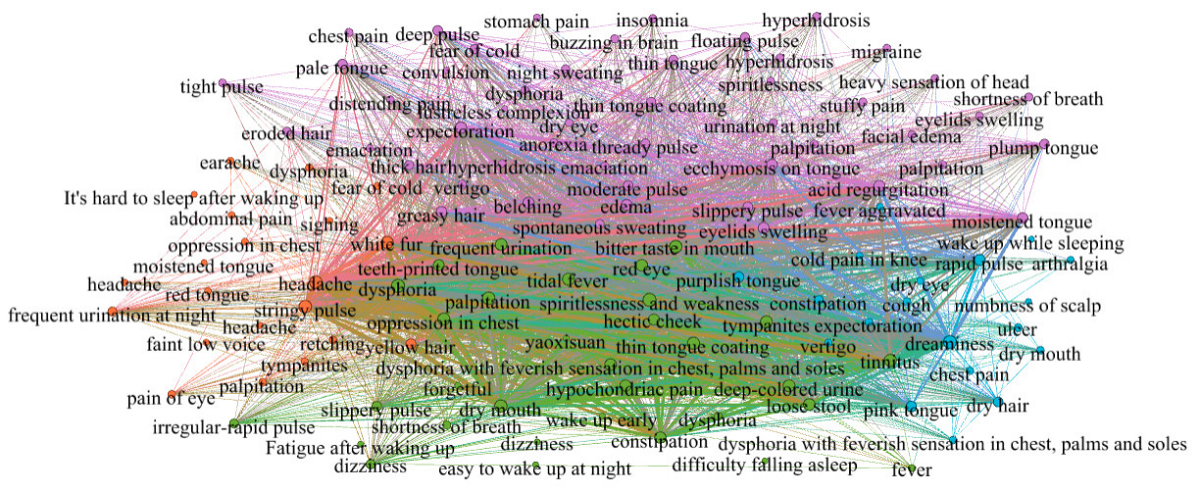
Symptom communities (d=164 and c=4).

**Figure 13:**
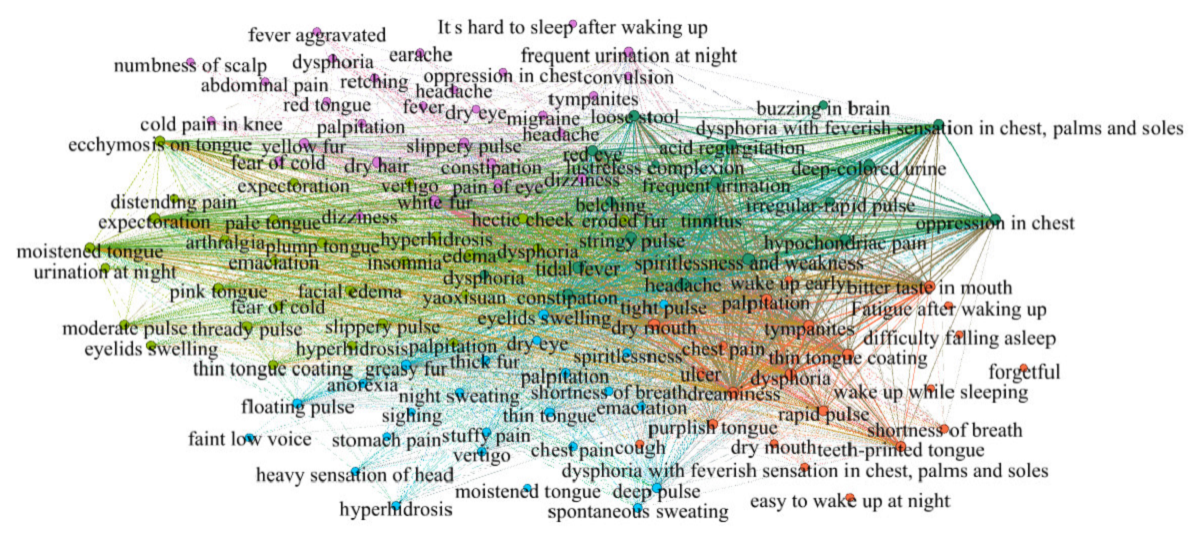
Symptom communities (d=164 and c=5).

The obtained symptom communities with different embedding dimensions and cluster numbers are shown in Figures 10-13. In these networks, the size of nodes denotes the degree of importance of symptoms of insomnia to the network; that is, a larger node indicates that this symptom is more important to insomnia. The size of the edges represents the co-occurrence frequencies of any two symptoms in the records. The clustering result revealed the classic symptom clusters of insomnia.

Some core symptoms such as *dry hair* in Figure 10, *frequent urination* in Figure 12, and *oppression in chest* in Figure 13 do not appear very frequently among the main complaints of patients. In addition, with regard to the disease subtypes for personalized treatment of insomnia, insomnia symptoms were only divided into four categories based on Figure 10 and Figure 12, which are too simple and cannot reflect the complexity and changeability of symptom characteristics of insomnia patients. In Figure 11, this symptom network (Figure 2) is split into ﬁve communities using the spectral clustering algorithm, which are more identical to the clinical diagnosis, as follows.

- Community 1 (green): symptoms including *spiritlessness and weakness, wake up while sleeping, fatigue after waking up, easy to wake up at night,* and *dreaminess* are divided into a community with the core symptom *difficulty falling asleep*.
- Community 2 (purple): symptoms including *dry hair, constipation, palpitation,* and *abdominal distension* are divided into a community with the core symptom *hard to sleep after waking up.*
- Community 3 (blue): the symptoms including *bitter taste in mouth, dry eye, rapid pulse, emaciation, and moderate pulse* are divided into a community with the core symptom *soreness and weakness of waist and knees*.
- Community 4 (pink): the symptoms including *purplish tongue, ulcer, earache, oppression in chest,* and *dry mouth* are divided into a community with the core symptom *pulse string.*
- Community 5 (orange): the symptoms including *expectoration, night sweating, thin tongue, ﬂoating pulse,* and *dizziness* are divided into a community with the core symptom *tinnitus.*

## Discussion

### Principal Findings

In this study, we considered insomnia as a model condition, and explored the symptom distribution regularity using complex network and machine-learning approaches focusing on a node-embedding representation. We constructed the symptom network to reﬂect the hidden relationships between symptoms, and then identiﬁed the core symptoms using representative evaluation metrics of node centrality. Based on the symptom network, we trained the symptom vocabulary using the node-embedding technique. After clustering symptom embeddings using the spectral clustering algorithm, we acquired the insomnia symptom communities, which can reveal the symptom distribution rule. The core symptoms were identiﬁed using representative evaluation indices of node centrality such as degree centrality, closeness centrality, betweenness centrality, and eigenvector centrality.

The results showed that the core symptoms are *difficulty falling asleep*, *easy to wake up at night*, and *dysphoria and irascibility*. Clinical research demonstrates that these symptoms always appear in the diagnosis of insomnia, and the majority of patients with insomnia have these three symptoms. According to the diagnostic criteria of International Classification of Sleep Disorders-3 in the European guidelines for the diagnosis and treatment of insomnia [36], the diagnostic criteria of chronic insomnia are: *difficulty falling asleep, difficulty maintaining sleep, getting up early, unwilling to go to bed on time, and difficulty falling asleep without intervention from parents or caregivers*. The five core symptoms of insomnia that we obtained (Figure 11) are *difficulty falling asleep, easy to wake up at night, dysphoria and irascibility, forgetful,* and *spiritlessness and weakness*. We further discovered the related symptoms corresponding to the core symptoms such as *irritability, dryness of mouth,* and *sweating at night*, which are all derived from the same syndrome. These ﬁndings also indicate the main syndrome for different individual cases. Therefore, our results essentially match the diagnostic criteria for the core symptoms of insomnia.

After training the node embeddings in the symptom network using the Skip-Gram model with different embedding dimensions (128 and 164), we acquired the different symptom embedding representations. We then clustered these symptom embeddings using the spectral clustering algorithm with different cluster numbers (4 and 5), and obtained four and ﬁve symptom communities, respectively. By comparing the experimental results with different dimensions and cluster numbers, we found that the clusters of insomnia symptoms are more identical to those in clinical practice and the results from previous studies when the dimension of the Skip-Gram model was 128 and the number of clusters in the spectral clustering algorithm was 5. Thus, the network shown in Figure 11 can reflect the distinct clinical symptom characteristics of insomnia, and each community is significantly heterogeneous, which will be helpful to evaluate the condition and guide individualized treatment.

### Limitations

To best evaluate the results of core symptom identiﬁcation or symptom clustering, we have simply presented the conclusion based on the symptom network structure analysis, evaluation metrics of node centrality in a complex network, and the similarity of symptom embeddings. The results were derived from objective calculations using machine-learning approaches. We also referred to the professional suggestions from clinicians working on insomnia, published manuscripts, and guideline for the diagnosis and treatment of insomnia. Because there is still no standard category for each symptom in TCM, the accuracy of the results remains to be verified.

### Conclusions

In the clinical practice of TCM, the symptoms of insomnia patients with different syndromes are different. Therefore, research focused on the identiﬁcation of core symptoms, syndromes, and their corresponding symptoms has signiﬁcance for the clinical diagnosis and treatment of insomnia. By using complex network and machine-learning approaches, specifically node-embedding and the spectral clustering algorithm, we constructed the symptom-weighted network model representing the relationships underlying the different symptoms. The insomnia symptoms were divided into ﬁve communities according to their distinct clinical characteristics. Multiple interrelated symptoms were frequently observed in the same community, reflecting the fact that different symptoms are derived from the same syndrome. These results can provide meaningful symptom associations, which can help physicians to ﬁnd the most signiﬁcant content and regularity from complex symptom relationships.

A similar diagnosis of symptoms appeared in a report by the Committee of the American Academy of Sleep Medicine [37]. Overall, the establishment of different communities can help to explore meaningful symptom associations, which can provide an intuitive understanding of the corresponding basic pathogenesis for physicians. Further, these results clarify that the methodologies used in this study can effectively and accurately ﬁnd hidden relationships between symptoms for insomnia. These methodologies can filter unimportant symptoms and obtain meaningful symptom correlations and associations, which will help physicians to find the most important core content from complex symptom relationships. The trained insomnia symptom embeddings can be used in additional research as a basic dataset. With further development, similar approaches can be used to explore the symptom distribution regularity for the diagnosis and treatment of other diseases.

## Abbreviations

EMR: electronic medical record
TCM: traditional Chinese medicine
